# Method of Stamping the Progression of a Beverage End Rivet of a Thinner Sheet of AW-5182 Alloy

**DOI:** 10.3390/ma16186244

**Published:** 2023-09-16

**Authors:** Mariusz Łucarz, Michał Jędrychowski

**Affiliations:** Faculty of Foundry Engineering, AGH University of Science and Technology, Reymonta 23 St., 30-059 Krakow, Poland

**Keywords:** beverage end, stamping, AW-5182 alloy, PAM STAMP simulations, quality tests

## Abstract

This paper presents a new solution for shaping the rivet progression of a beverage end. The classic method uses three operations to press the cylindrical rivet using 0.208 mm and 0.203 mm thick sheets. The increasing demand for aluminium alloys is prompting measures to make more efficient use of this raw material. One possible solution is to produce packaging from ever thinner sheets. This requires the design of new tooling and the preparation of an appropriate technological process. A method has been developed to stamp a hexagonal-shaped rivet from 0.200 mm thick sheet metal, increasing the number of stamping operations to four. The proposed method was verified through a numerical analysis using the PAM STAMP 2022.0 software package. It was found that for appropriately shaped tools, sheet thicknesses of the stamped component could be achieved that were not less than those for the currently used technology, thus eliminating any possible break in the material structure. Suitable tools and experimental stamping tests were carried out for the developed process. In the simulations, the material Al5182_iso_Xmm was adopted from the programme database, while the experiments were performed on a laboratory press using AW-5182-H48 sheets with a thickness of 0.200 mm. The purpose of the study was to determine the validity for the proposed method of forming the rivet of the beverage end.

## 1. Introduction

For economic and environmental reasons, the modern economy is moving towards the production of closed cycle products, as well as the production of various types of packaging that can be reused many times. A beverage can composed of aluminium alloy meets this criterion. The growing demand for the raw material from which the can is made, with the phasing out of plastic packaging, is prompting manufacturers to adopt the best possible management of the material in circulation. One way to manage it efficiently is to try to produce beverage ends from thinner sheets. A method to cope with this, while maintaining product functionality without defects, is the development of appropriate stamping technology and tools. For the sheet thicknesses currently used to stamp beverage ends, three operations are used, in which, if a thinner sheet thickness is used, excessive thinning of the sheet occurs, as well as disruption of the material structure. Therefore, a study has been carried out to implement the stamping process in such a way that the sheet metal with a smaller thickness after the operations performed does not undergo greater thinning relative to the current technology used.

One of the leading materials in the extrusion process is aluminium alloys, which have several applications arising from their wide range grouped into series from 1000 to 8000, with different alloy additives ranging from silicon (Si) to magnesium (Mg) in each group [1,2,3,4]. Aluminium alloys are used in unrelated industries because of their unique properties; therefore, they are used in the marine, automotive, aerospace, or food industries.

Aluminium alloys of the 5000–7000 group are used in areas where their anticorrosion properties are useful. Historically, corrosion-resistant aluminium alloys have been used to build police boats [5]. The corrosion resistance of aluminium alloys is an important feature for food storage. Initially, aluminium alloys in the food industry found a general use [6], until their use today in beverage cans [7,8,9]. Important parameters of aluminium alloys are their ability to be stamped as presented in the publication [10]. An important area of use of aluminium alloys is the automotive industry, which takes advantage of another feature of aluminium alloys, i.e., their lightness, which significantly reduces the weight of the product [11,12,13]. The lightness of the material is also important in the manufacture of aircraft components [14,15].

Aluminium sheets to stamp various types of components are divided into degrees of reinforcement appropriate for the purpose [1,9]. In the food industry, beverage cans are made from aluminium alloys, consisting of components: can (AA3104-H19), end (AW-5182-H48), and key (AW-5182-H48). The history of beverage packaging manufacturing dates to 1935 [16]. At that time, a three-piece tinplate can was developed. The first two-piece aluminium can (can and end) was developed by the Adolph Coors Company in Golden in 1958 [16], with a thickness of 0.762 mm.

The engineering and manufacturing of lightweight cans that maintain structural integrity is a complex process. The various stages of stamping are outlined in the paper [17]. Canning technology is related to the chemistry of can coatings, which are used to protect food and beverages from the metal surface (to prevent taste deterioration) and the metal surface of food products (to protect against corrosion) [18]. In aluminium cans, a thin layer of Al_2_O_3_ forms when exposed to air or water. However, this layer shows solubility at both low and high pH levels of stored substances and at high NaCl concentrations [19]. In a closed can, the presence of oxygen is limited, and, without the oxide layer, corrosion of the aluminium occurs. Thus, without organic coating, the durability of an aluminium can is insufficient [20].

The beverage can has undergone many design changes over time, adopting its current shape and weight, which represents a great advance in terms of the design and production technologies developed [7,21]. Today’s choice of nonferrous metal packaging is determined primarily by the lightness of the material, good strength properties, ease of recycling of the alloy used or disposal of the additional components that comprise the can [7,8,22]. In the recycling process, incineration is the most used method to remove the manufactured protective coatings. To reduce CO_2_ emissions, research is being conducted to develop environmentally friendly methods. One such method is presented in the article [23].

The stamping process involves giving a specific profile to a component through the use of shaping tools such as a die and a punch [16,24,25,26,27]. Stamping can be carried out using cold or hot methods [4]. Cold stamping is the predominant method for forming aluminium alloy sheets due to its high production efficiency and low cost [28]. 

Some aluminium alloys are characterised by the possibility of deep stamping using an intermediate operation such as over stamping (thinning the thickness of the extruded material) [29].

Currently, tool builders and production process technologists have an easier task due to the possibility of using computer simulations. Dedicated programmes for these activities are ABAQUS, ETA/DYNAFORM, and PAM STAMP. In publications [30,31], the authors address defects that arise during the stamping process as a result of computer simulations with the ABAQUS programme and ETA. An important advantage of using the PAM STAMP programme is that it supports the stamping process by eliminating manufacturing risks and possibly related production costs in advance [32]. The authors of the publication [33] point to the PAM STAMP programme as a good tool for FEM analysis and evaluation of the stamping process.

In a paper [34], the authors also found that process simulation with the PAM STAMP programme is a reliable tool for analysing and improving the hot and warm forming process of aluminium alloys (AW-5182). Simulations make it possible to reduce the preparation of subsequent tool prototypes by trial and error in the preparation of new solutions, by pointing out possible design flaws in the design stage [29,35]. One of the most common problems encountered during the stamping process is a break in the material structure of the sheet metal due to stalling. This results in a crack or too much material thinning, so that so-called micro leaks can occur [26,30,36,37,38]. To avoid this, it is important to choose the edges of the right parameters for the punch and die, as shown in the work [34] when shaping rectangular ribs in a heat exchanger.

The food industry uses plastic packaging; however, at present, for environmental reasons, biodegradable or easily recyclable materials are preferred. As a result, the demand for materials that can function in a closed loop economy has increased significantly. Such products include aluminium cans. Alloys from the 5xxx series (AlMgMn–AW-5182) are used to manufacture beverage ends [39]. The increasing demand for aluminium, to the extent described, is prompting measures to make better use of this raw material. One possible solution is to produce packaging from ever thinner sheets. This requirement can be met by designing new tooling and preparing a suitable technological process. Simulation programmes used for sheet metal stamping are helpful in this regard. Without making expensive prototypes, it is possible to verify, as a result of numerical simulations, whether the designed tooling will meet expectations when forming a specific package from sheet metal of decreasing thickness. At the same time, as pointed out in [40], excessive reduction in the thickness of the sheet during rolling makes the manufacturing process unstable, and this leads to variations in the sheet thickness and, consequently, the can. 

An important aspect of the simulations performed is the determination of the optimum shapes and number of tools that will guarantee the realisation of the process when using a 0.200 mm thick sheet.

## 2. Materials and Experiment

### 2.1. Material

The production of the beverage ends is carried out with AW-5182 alloy. The chemical composition of the material used in EN 573-1 [41] is shown in Table 1. The physical properties of the alloy analysed used in the tests are shown in Table 2.

The strength properties of smooth aluminium sheets (according to EN 485-2 [42]) for thicknesses in the range 0.200–0.500 mm and reinforcement H48 (reinforced by rolling and painted or varnished–hard reinforcement condition) are shown in Table 3.

### 2.2. Shaping the Beverage End Rivet Progression

**Figure 1:**
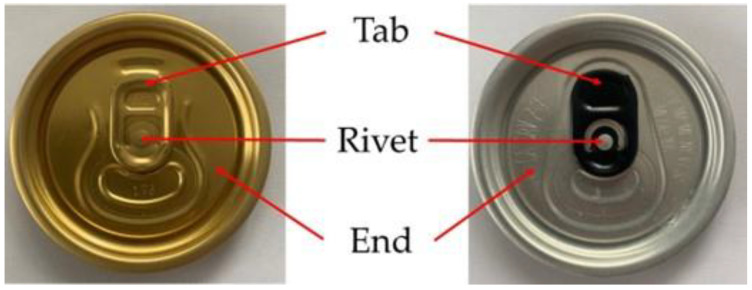
The components of a beverage end.

One of the embossed elements in the production of a beverage end is the rivet to attach the key that enables the can to be opened. The individual components of the example final product are shown in Figure 1.

The process of shaping the rivet progression (successive stamping steps to give a specific shape) for the currently used sheet thicknesses of 0.208 mm and 0.203 mm is carried out in 3 cycles. The rivet is currently shaped in production on the basis of a circle. Economic considerations, as well as the increasing demand for aluminium alloys, have led to the notion of trying to produce a beverage can from a thinner sheet thickness of 0.200 mm, which can lead to excessive thinning of the material and, as a consequence, to a break in the material structure or the formation of micro-leaking. In order to prevent this, a decision was made to change the rivet progression technology to 4 stamping operations (additional embossing). At the same time, a rivet shape with a hexagonal base was adopted. This shape required the introduction of appropriate radii on the individual edges of the designed rivet.

During the simulation, the radii were changed:

R_1_–the radius between the end and the side of the shaped rivet;
R_2_–the radius between the side of the shaped rivet and the top surface;
R_3_–the radius between the sides of the shaped body with a hexagonal base.

**Figure 2:**
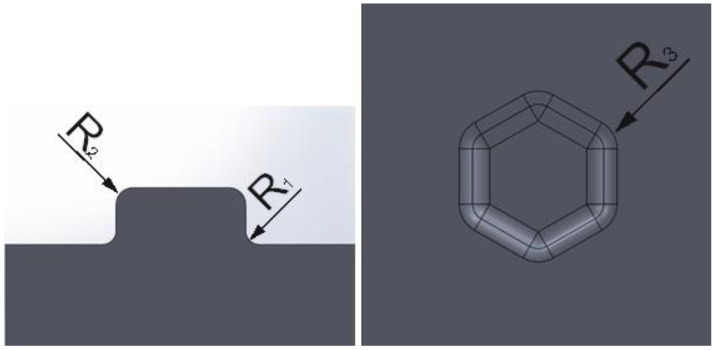
Designation of the radii used in tool shaping.

Figure 2 shows the individual radii on the example stamp.

**Figure 3:**
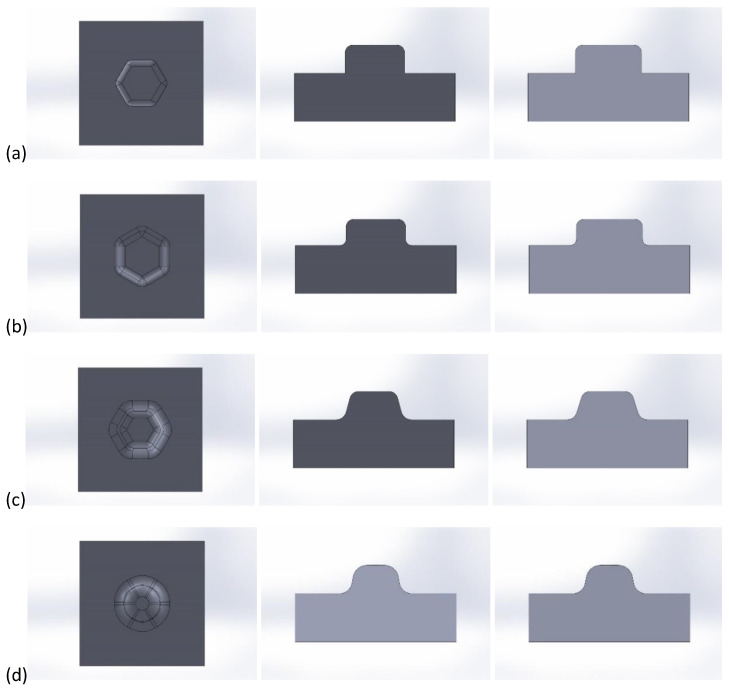
View of the individual punch shapes prepared for stamping: (**a**) shape 1, (**b**) shape 2, (**c**) shape 3, (**d**) shape 4.

Figure 3 shows the shapes of the different stamps used in the numerical simulations in PAM STAMP, with different values of the radii R_1_, R_2_, and R_3_ applied, from the smallest values to increasingly larger values.

The selection of the appropriate tooling for the stamping process began with a sheet metal shaping in PAM STAMP. This procedure eliminates the construction of physical tooling by trial and error. Suitable tool set shapes (punch and die) were created using CAD (SolidWorks 2021–2022) and imported into the simulation programme, selecting a suitable mesh to enable the best accuracy of representation of the designed components. For the numerical simulations, the material Al5182_iso_Xmm was adopted from the database available in the software.

In the simulation studies, two parameters of the aluminium sheet were analysed: the thickness and the thinning of the sheet after stamping. From the point of view of airtightness and strength, these are important indicators, especially when it comes to the storage of pressurised beverages in thin-walled cans.

A set of stamping tooling was made by machining based on the simulations performed and the final solution adopted.

The prepared tooling was placed on a laboratory press and the prototypes of the rivet progression were moulded.

### 2.3. Testing Methods

Strength tests were carried out to determine the basic parameters of the metal sheets of different thicknesses used to stamp beverage ends. The results presented in the publication are for sheets from sample suppliers. The tests were carried out to check that the sheets met the parameters according to the standard. The mechanical properties of the aluminium sheets were tested on an MTS EXCEED model e43.

**Figure 4:**
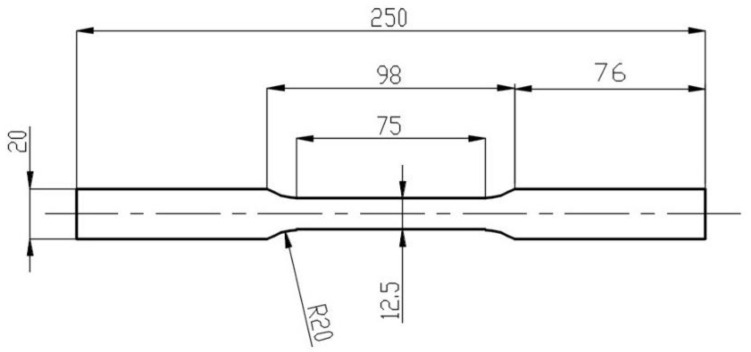
Dimensions of the strength test specimen.

**Figure 5:**
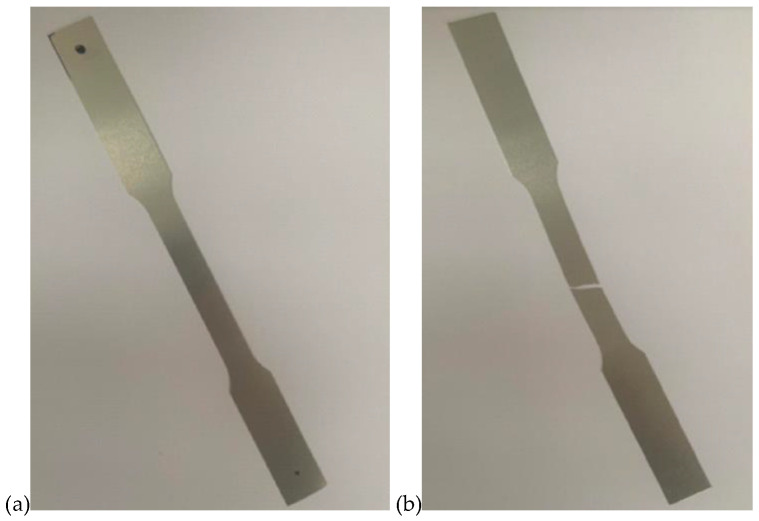
Example test specimen: (**a**) before testing, (**b**) after breaking.

The tests were carried out on samples dedicated to the machine. The dimensions of the specimens are shown in Figure 4. Twenty-five specimens were prepared for each thickness of the sheet for strength testing. The pre-test and post-test output sample is shown in Figure 5.

**Figure 6:**
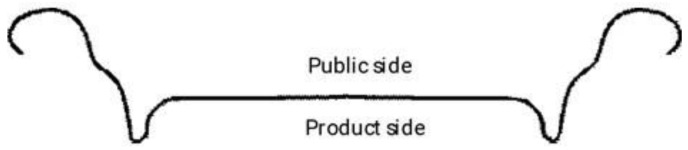
Shape of the beverage end.

The quality of the ends produced is subjected to several tests during the production process. The first is to test the resistance to pressure exerted (e.g., gas from a beverage) on the produced part. Figure 6 shows an example of a cross section of a beverage end.

The pressure resistance test involves placing the component with the “product side” (beverage contact) in the die of the measuring device and applying the appropriate pressure. In the production of beverage cans, it is required that the component in its fresh (manufactured) state can withstand the pressure as specified. 

Additionally, the leakage of the manufactured end is tested using two methods:

− light beam test;
− micro-leakage test (pressure).

The first test consists of applying a strong light beam to the “product side” (the surface in contact with the beverage), which, in the event of small breaks in the material, pierces through the aluminium part. The end is then automatically rejected during the production process. 

**Figure 7:**
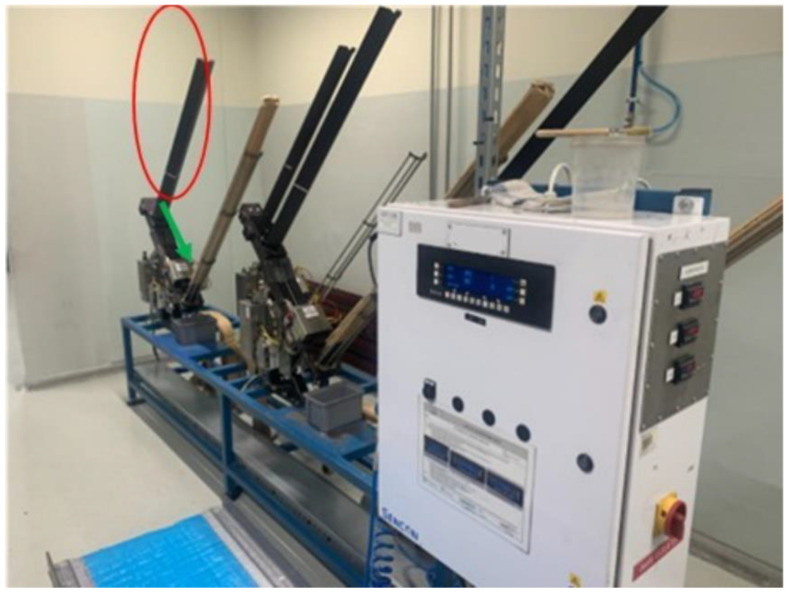
Beverage end leak tester.

The latter test is carried out on finished ends when a leak is detected on a selected batch of finished product during quality control (selective measurements are carried out several times during the working day). Automatically, the entire production batch is tested in the laboratory. Figure 7 shows the leak testing device.

The red colour in Figure 7 indicates the tray in which the ends for the test are placed and then, following the direction of the green arrow, enter the measuring matrix.

**Figure 8:**
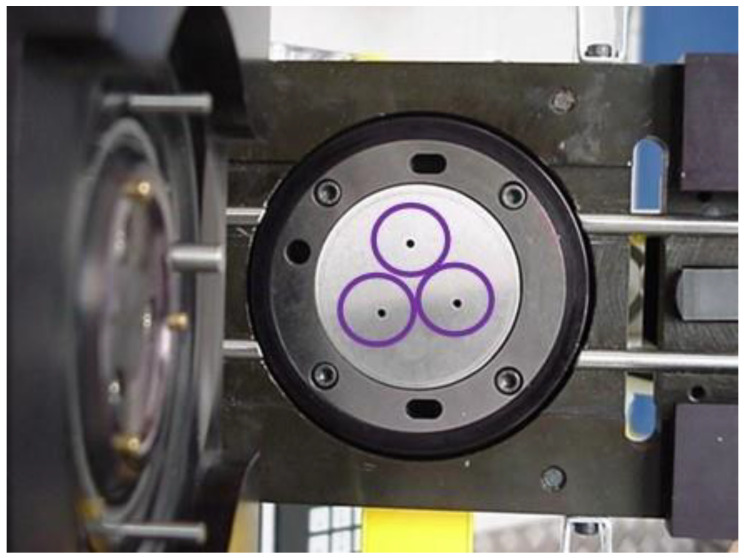
Measurement matrix for leak testing.

In the die, a specific pressure is applied to the side of the end on the purple product side through the holes (Figure 8). If the end has a leak-related defect, the pressure is recorded through the top of the die, and the defective part is rejected for scrap. Ten ends with a new rivet progression were tested.

Images of the extruded ends were taken using a Keyence VHX-7000 series digital microscope (Keyence Ltd. HQ & Laboratories, Osaka, Japan) shape registration to evaluate the shape obtained. A high-precision 4 K microscope captures images of 3D objects in high resolution. High-precision 3D imaging is made possible by KEYENCE’s DFD 2.0 algorithm. The High Dynamic Range (HDR) imaging function achieves high colour gradation by capturing multiple images at different exposure times. This allows for high precision and contrast of the observed image.

**Figure 9:**
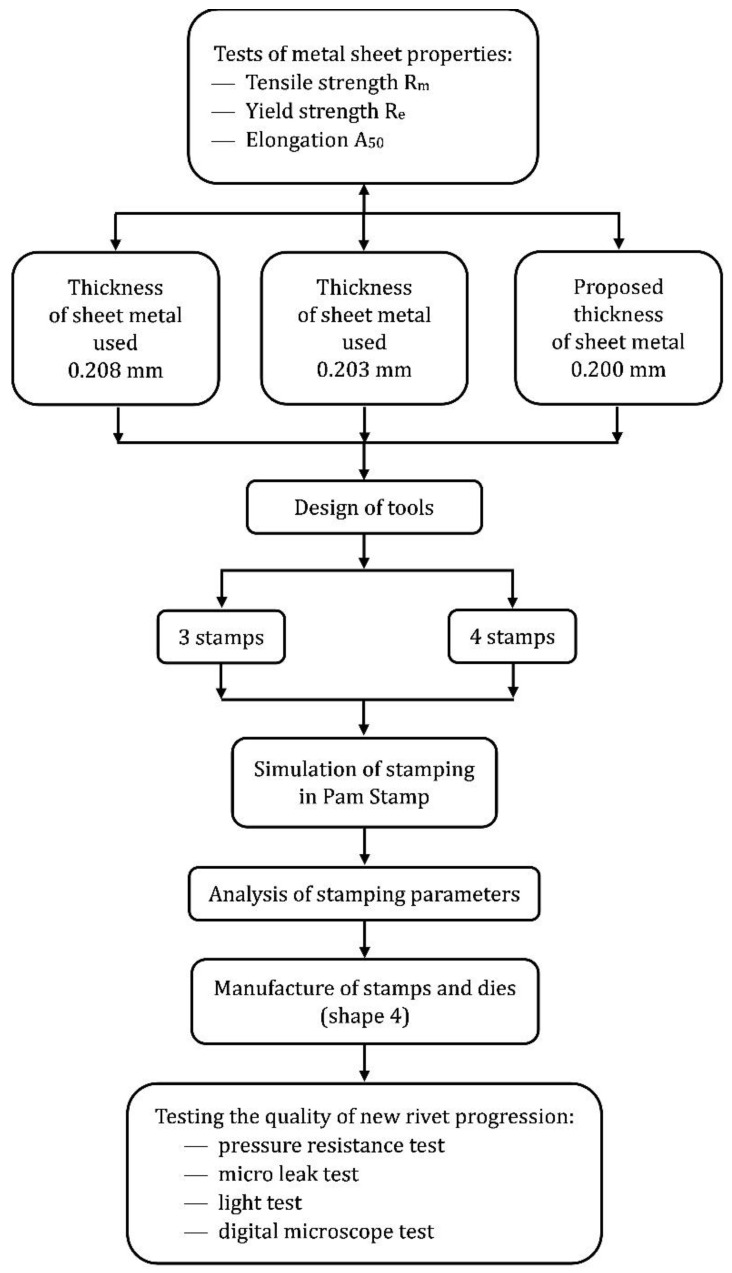
Flowchart of sheet metal stamping tests and analysis.

The programme of the full experiment performed is shown in Figure 9.

## 3. Results

Tensile tests were carried out on three sheet thicknesses to produce beverage ends composed of the alloy AW-5182: the currently used 0.208 mm and 0.203 mm; and the one planned for introduction with a thickness of 0.200 mm. The main mechanical parameters determined in the tensile tests were the yield strength R_e_, the tensile strength R_m_, and the percentage of elongation A_50_.

The tests performed are for sheets that were on the production line at the time of the article. At different times, the mechanical properties of the analysed sheets may be different from those presented. 

Table 4 shows the results of the strength tests on 0.208 mm thick sheet metal performed on 25 producer 1 [0.208 (1)], and Table 5 of producer 2 [0.208 (2)]. The purpose of the tests performed was to verify, in terms of strength parameters, the quality of the sheet metal depending on the source of purchase.

**Figure 10:**
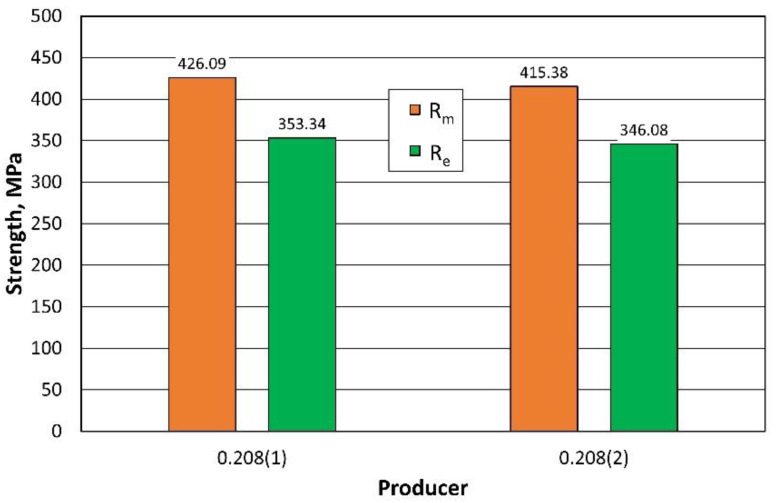
Comparison of the strength parameters of 0.208 mm thick sheets from 2 producers.

Figure 10 compares the strength results obtained for sheets of the same thickness from two suppliers. The results are different, but both sheets meet the criteria of the adopted EN 485–2 standard (Table 2) with regard to yield and tensile strength.

**Figure 11:**
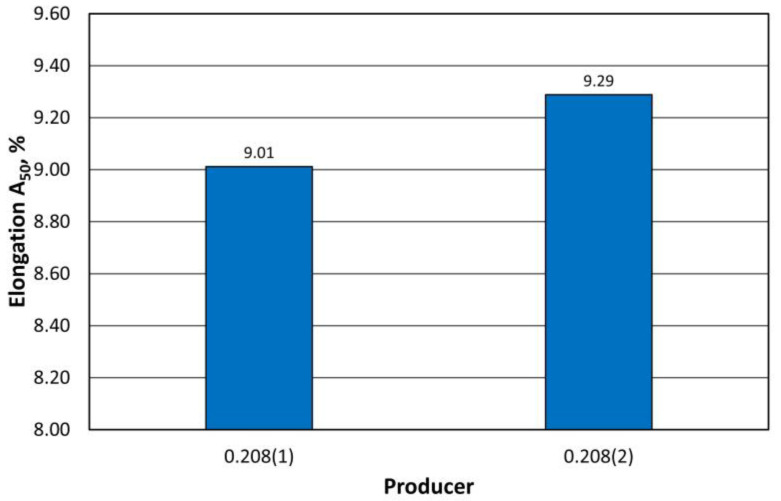
Comparison of the A_50_ elongation of 0.208 mm thick sheets from 2 producers.

The A_50_ elongation also meets the standard. A comparison of the results obtained is shown in Figure 11.

In the following part of the study, measurements were carried out for sheets with a smaller thickness. The results for the 0.203 mm sheet are shown in Table 6, while the results for the 0.200 mm sheet are shown in Table 7.

**Figure 12:**
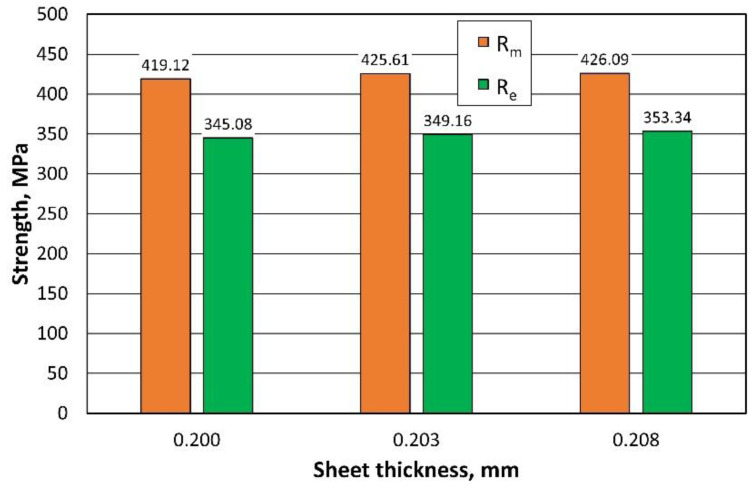
Comparison of the strength parameters of sheets with different thicknesses.

Figure 12 summarises the results of the mean values of the strength measurements taken as a function of the thickness of the sheet. The changes in strength are negligible and, as expected, the larger the cross section of the specimen, the higher the strength.

**Figure 13:**
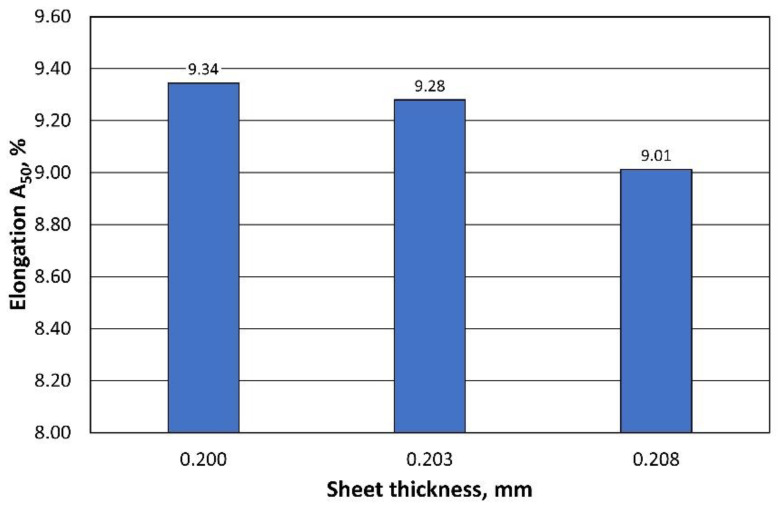
Comparison of the A_50_ elongation of sheets with different thicknesses.

The elongation of A_50_, on the other hand, decreased with increasing sheet thickness, as shown in Figure 13.

The tests performed on the mechanical parameters of the sheets were the starting point for analysing the stamping process of the beverage end rivet. 

**Figure 14:**
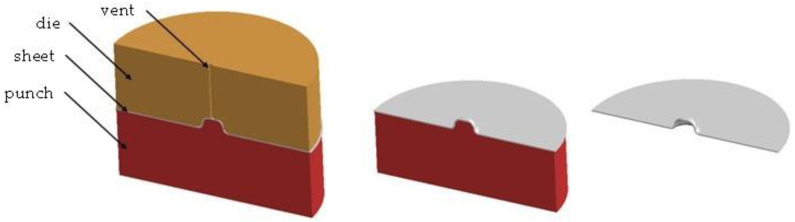
Example view of the die and stamp used in the simulations using PAM STAMP software and the finished product.

**Figure 15:**
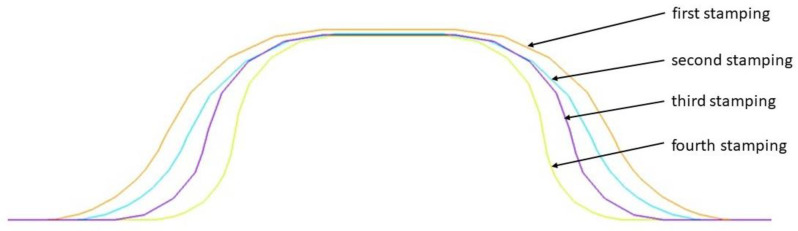
Rivet shaping progression for 4 operations.

Suitable stamping elements were prepared. Figure 14 shows an example of the appearance of the developed stamping tools. Figure 15, on the other hand, shows the progression of rivet forming in four operations.

Numerical calculations were performed for the prepared 32 sets of stamping tools according to their shapes, as shown in Figure 3.

The following relationships in radius values occurred between the different tool shapes (the first index refers to the radius; the second index to the tool shape):

R_11_ < R_12_ < R_13_ < R_14_

R_21_ = R_22_ < R_23_ < R_24_

(R_31_ > R_32_) < R_33_ < R_34_

The results of the minimum sheet thickness (Thk_min_) and maximum thinning (Thn_max_) of all 24 simulations performed for the three and four stamping operations, analysed according to the sheet thickness, are summarised in Table 8. When using three tools, the first stamping is omitted.

**Figure 16:**
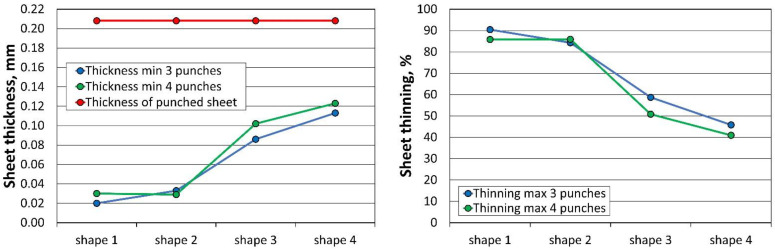
Summary results of rivet progression simulations performed with the PAM STAMP programme for a 0.208 mm thick sheet.

**Figure 17:**
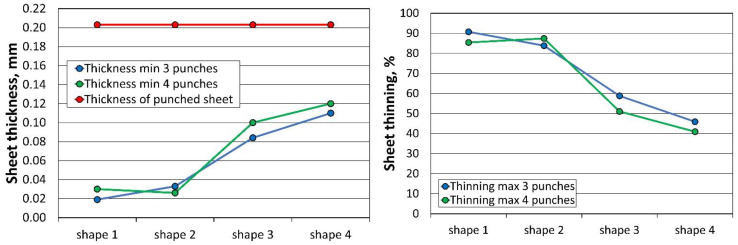
Summary results of rivet progression simulations performed with the PAM STAMP programme for a 0.203 mm thick sheet.

**Figure 18:**
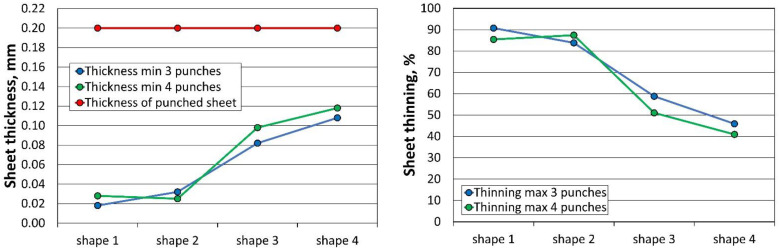
Summary results of rivet progression simulations performed with the PAM STAMP programme for a 0.200 mm thick sheet.

The profile radii of the fabricated tools have a significant impact on the sheet metal stamping to obtain the correct shape of the components. This is confirmed by the numerical simulations performed. For all the sheet thicknesses for tools according to shapes 1 to 3, the results of the computer simulations showed excessive thinning of the material, as illustrated in Figure 16, Figure 17 and Figure 18. Sufficiently large radii R_1_, R_2_, and R_3_ of the adopted shape 4 of the stamped rivet created conditions for obtaining sheet thicknesses at the cross section that can guarantee the production of a correct rivet. The simulations carried out also showed how important the relation between the radii of the stamped component is in the example realised. The use of four dies for all sheet thicknesses favours an increase in the minimum thickness Thk_min_ of about 0.009 mm for tools, according to shape 4.

**Figure 19:**
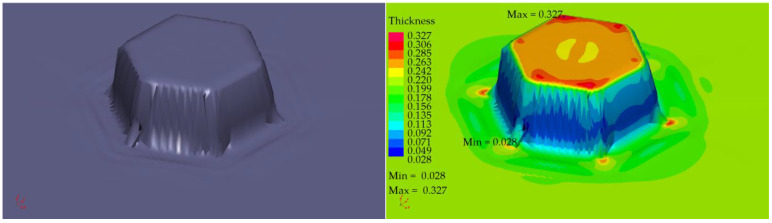
Simulation results for shape 1–0.200 mm thick sheet, 4 stamping dies.

In the case of the stamping of 0.200 mm sheet metal using the tools designed according to shape 1, there was a high tendency to corrugate the sheet metal as well as to crack the stamped material, as illustrated in Figure 19.

**Figure 20:**
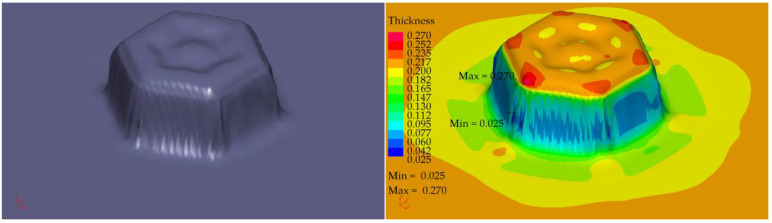
Simulation results for shape 2–0.200 mm thick sheet, 4 stamping dies.

**Figure 21:**
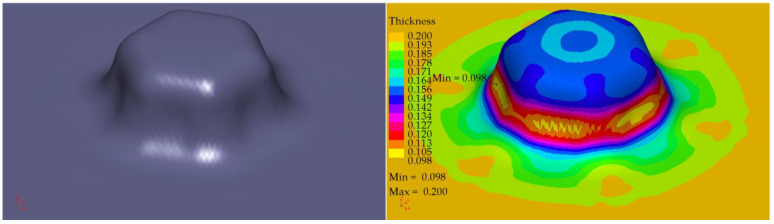
Simulation results for shape 3–0.200 mm thick sheet, 4 stamping dies.

**Figure 22:**
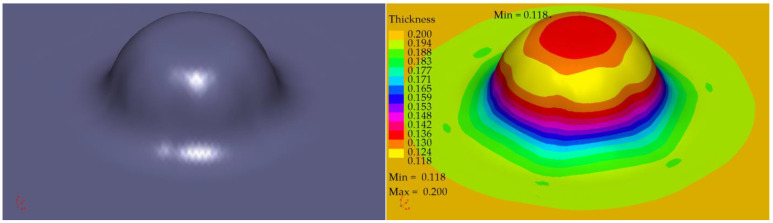
Simulation results for shape 4–0.200 mm thick sheet, 4 stamping dies.

Tool shape 2 is also unsatisfactory, causing excessive thinning of the sheet on the cross-section (Figure 20). The use of tool shape 3 does not cause wrinkling of the material (Figure 21); however, there is thinning of the sheet in a place that poses a real danger of the structure cracking during the riveting process. Shape 4 of the tools creates the conditions for the material to be free flowing, so that the sheet thinning is located in a safe area for riveting (upper part of the shaped rivet), which reduces the risk of a crack in the material structure during riveting (Figure 22). 

**Figure 23:**
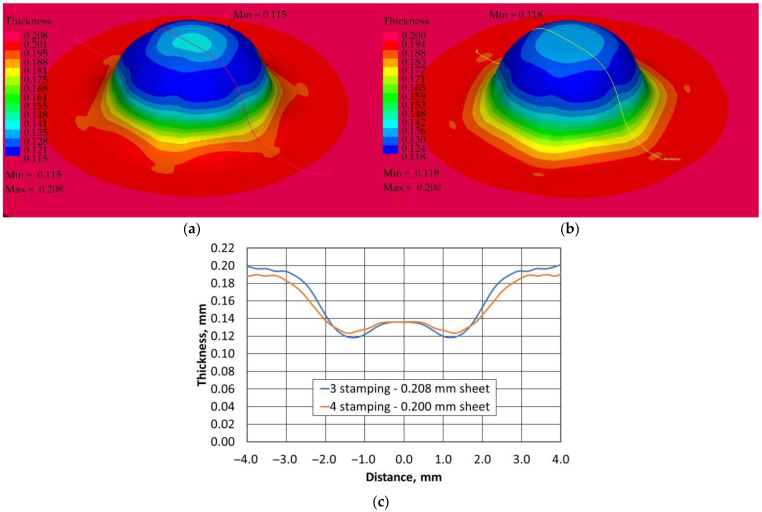
Variation in thickness of the pressed sheet depending on its thickness and the number of tools used: (**a**) 3 stamping dies, (**b**) 4 stamping dies, (**c**) variation in thickness in the indicated section.

The introduction of an additional four stamping operation, which was performed first, for the 0.200 mm sheet, had the task of stamping the sheet so that the final thickness was not less than for thicker sheets performed in three stamping operations. Figure 23 illustrates the change in the cross-section of a 0.208 mm sheet stamped in three operations and a 0.200 mm sheet in four operations. The use of an additional stamping step for the 0.200 mm sheet resulted in smaller thinning of the thinner sheet than that of the currently used thicker sheet. At the same time, the thinner sheet had a higher thickness at the highest stamping point. If we compare the Thk_min_ thickness of 0.115 mm obtained for three stamping dies for 0.208 mm sheet metal with the Thk_min_ thickness of 0.118 mm for four stamping dies for 0.200 mm sheet metal, the computer simulations indicate less thinning of the thinner sheet metal, which was the intention of the experimental work undertaken.

**Figure 24:**
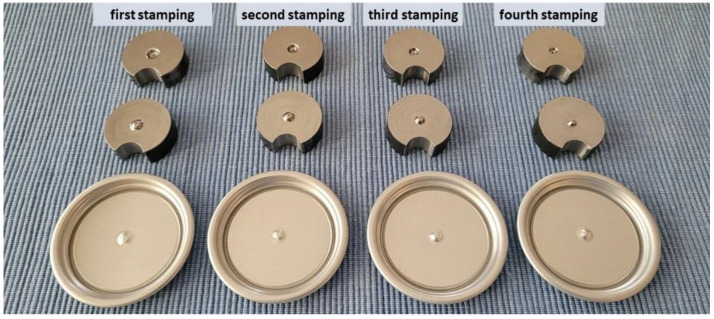
Stamping tools and stages of completion of the rivet progression on the end.

Calculations made with the PAM STAMP programme allowed the shape of the rivet progression tools to be selected for the 0.200 mm sheet, ensuring that the shaped part was produced without defects. Figure 24 shows the tools produced and the various stages of shaping the rivet on the end.

A pressure test of the produced beverage ends was carried out. The test result for all of them was positive.

**Figure 25:**
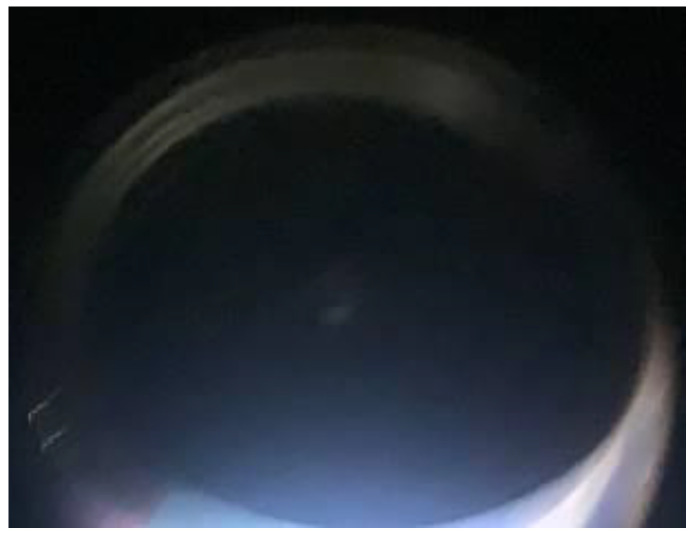
Example photograph of a 0.200 mm beverage end after four stamping operations during a light beam test.

A light beam test was also performed. Figure 25 shows an example of a photo of a tested beverage end. Again, no defects were found in the discontinuity of the material structure.

**Figure 26:**
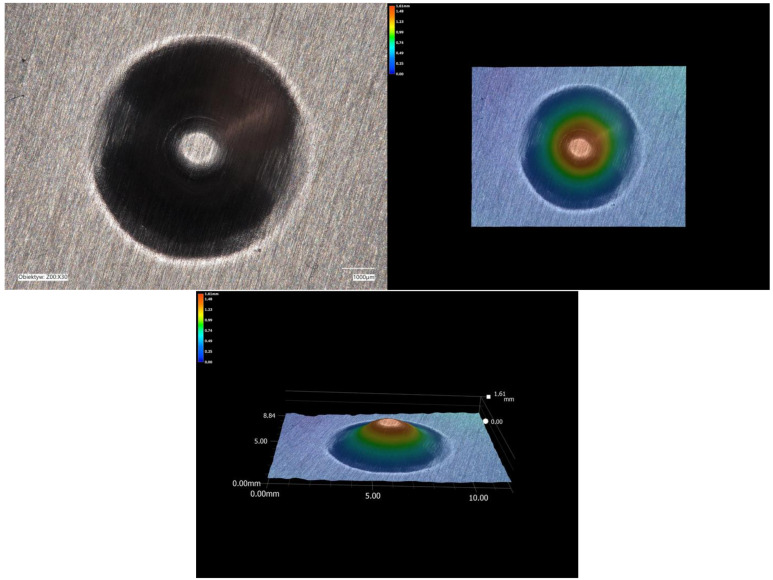
Mapping the shape of a beverage end rivet using an optical microscope with a 3D function.

As the stamped shape of the beverage end rivet is small, its correct mapping was therefore verified using a digital microscope with 3D imaging. An image of the stamping performed is shown in Figure 26. The operations performed resulted in the expected shape.

An additional fourth stamping operation will not affect the efficiency of the production process, since this rivet shaping step can be combined with the stamping of another element on the beverage end (e.g., signatures on the end, promotional codes, or others, which once occur and other times do not, but a sufficient number of presses are available in the process line). Estimates show that the cost of producing additional tools will pay for itself after four days of operation of a single press. One 0.200 mm thick sheet of AW-5182 aluminium alloy uses 87 kg less material to produce the same number of ends. On the other hand, from the saved amount of material it is possible to additionally produce about 45,000 more beverage ends, assuming that one end weighs about 1.9 g.

## 4. Conclusions

The tools designed, the calculations performed, and the technological tests enable the following conclusions to be drawn.

1. Simulations performed for different shapes of stamping dies indicate that one of the most important factors of the stamping process is the geometry of the tools that form the stamping with the expected final thickness and thinning of the sheet.
2. The transition radii between the different surfaces, i.e., the shape of the stamping tools, should ensure the free flow of the stamped material. The use of sharp-edged tools promotes breakage of the continuity of the stamped material.
3. Stamping tools of a closed shape, such as the rivet of a beverage end, require venting to evacuate air to limit the deformation of the stamping during forming.
4. The introduction of four additional stamping operations for the sheet metal in the rivet-forming progression created the conditions for obtaining a properly shaped component, as expected. The thinning of a 0.200 mm sheet is less than for a 0.208 mm sheet stamped with three stamping operations.
5. As computer simulations have shown, the designed tools can also be used successfully to shape the rivet progression for the sheet metal currently in use. No retooling of the presses will be required when changing sheet thicknesses.
6. A concept has been developed to form the progression of the rivet of a beverage end from the 0.200 mm thick sheet alloy AW-5182-H-48.
7. An important element of the stamping process is the reproducibility of the mechanical properties of the material, which is difficult due to the production process realized by different suppliers.
8. The development of a suitable technology for the production of the beverage end rivet creates the conditions for the use of thinner sheets, and thus better use of the material with the increasing demand for it.

## Figures and Tables

**Table 1:** Chemical composition of alloy AW-5182 according to EN 573-1.

Mg [%]	Mn [%]	Fe [%]	Si [%]	Cu [%]	Zn [%]	Cr [%]	Ti [%]	Other Each [%]	Other All [%]	Al [%]
4.00–5.00	0.20–0.50	≤0.35	≤0.20	≤0.15	≤0.25	≤0.10	≤0.10	≤0.05	≤0.15	rest

**Table 2:** Physical properties of alloy AW-5182.

Density [g/cm^3^]	2.7
Modulus of elasticity E [MPa]	71,000
Longitudinal elastic modulus G [MPa]	26700
Poisson’s ratio	0.33
Solidification temperature [°C]	585
Pour point [°C]	640
Specific heat in 20 °C [J/kg∙K]	902
Thermal coefficient of expansion in 20 °C [μm/m∙K]	24

**Table 3:** Strength properties of the AW-5182 H48 alloy.

Tensile strength R_m_ [MPa]	370–430
Yield strength R_e_ [MPa]	330–390
Elongation A_50 min_ [%]	4

**Table 4:** Results of strength tests on 0.208 mm thick sheet—producer 1.

Parameters	F_max_ [N]	R_m_ [MPa]	R_e_ [MPa]	A_50_ [%]
Max	1119.20	430.40	360.00	9.70
Min	1098.70	422.60	348.40	8.10
Average	1107.82	426.09	353.34	9.01
Standard Deviation σ	4.77	1.83	3.01	0.39

**Table 5:** Results of strength tests on 0.208 mm thick sheet—producer 2.

Parameters	F_max_ [N]	R_m_ [MPa]	R_e_ [MPa]	A_50_ [%]
Max	1089.30	419.00	355.40	11.10
Min	1072.40	412.50	335.00	7.90
Average	1079.93	415.38	346.08	9.29
Standard Deviation σ	4.68	1.79	6.20	0.79

**Table 6:** Results of strength tests on 0.203 mm thick sheet.

Parameters	F_max_ [N]	R_m_ [MPa]	R_e_ [MPa]	A_50_ [%]
Max	1071.80	428.70	355.20	10.80
Min	1056.20	422.50	338.70	8.10
Average	1064.04	425.61	349.16	9.28
Standard Deviation σ	4.68	1.88	3.67	0.67

**Table 7:** Results of strength tests on 0.200 mm thick sheet.

Parameters	F_max_ [N]	R_m_ [MPa]	R_e_ [MPa]	A_50_ [%]
Max	1074.80	423.60	355.70	10.60
Min	1053.10	415.00	337.80	7.80
Average	1063.87	419.12	345.08	9.34
Standard Deviation σ	5.15	2.09	3.84	0.64

**Table 8:** Simulation results for the third and fourth operations of the stamping process for the thicknesses of the analysed sheets.

Thickness [mm]	Shape	Shape 1		Shape 2		Shape 3		Shape 4	
	Parameters	Thk_min_ [mm]	Thn_max_ [%]	Thk_min_ [mm]	Thn_max_ [%]	Thk_min_ [mm]	Thn_max_ [%]	Thk_min_ [mm]	Thn_max_ [%]
0.208	3 punches	0.020	90.4	0.033	84.3	0.086	58.7	0.115	44.8
	4 punches	0.030	85.8	0.029	85.9	0.102	50.8	0.124	40.2
0.203	3 punches	0.019	90.7	0.033	83.8	0.084	58.8	0.112	44.9
	4 punches	0.030	85.4	0.026	87.4	0.100	51.0	0.121	40.2
0.200	3 punches	0.018	90.9	0.032	84.0	0.082	58.9	0.110	44.9
	4 punches	0.028	86.1	0.025	87.7	0.098	51.0	0.118	40.3

## Data Availability

The data that support the findings of this study are available from the corresponding authors (M.Ł.) upon reasonable request.
